# From green to red: metabolic reprogramming and bacterial community succession underpin cherry tomato fruit ripening and quality formation

**DOI:** 10.3389/fpls.2026.1843442

**Published:** 2026-06-08

**Authors:** Muzammil Hussain, Nazir Ahmed, Zhengzhou Yang, Xiaona Xie, Wenjing Xing, Hongzhu Su, Qingqing Peng, Zhengjie Zhu

**Affiliations:** 1 College of Agriculture and Food Engineering, Baise University, Baise, China; 2 Guangxi Key Laboratory of Biology for Mango, Baise, China; 3 College of Subtropical Characteristic Agricultural Industry, Baise, China

**Keywords:** aroma and flavor, cherry tomato, fruit microbiome, fruit quality, metabolomics, plant-microbe interactions

## Abstract

Tomato fruit ripening is accompanied by profound biochemical and microbial-associated changes that collectively shape fruit flavor, quality, and susceptibility to (a)biotic stresses. However, integrated insights into the co-varying effect of metabolic reprogramming and fruit-associated microbiome across ripening stages remain limited. Here, we employed a multi-omics approach to investigate stage-dependent shifts in the metabolome and bacteriome of cherry tomato fruits across three ripening stages: mature green, pink, and red ripe. Fruit quality analysis revealed a significant increase in soluble sugars, lycopene, and ascorbic acid from the green to the red stage. Untargeted metabolomics showed extensive metabolic reprogramming during ripening, characterized by the marked accumulation of lipids, amino acids, carbohydrates, terpenoids, and flavonoids in red ripe fruits, alongside a decline in defensive alkaloids such as tomatine. High-throughput 16S rRNA amplicon sequencing showed that bacterial diversity and community composition shifted significantly with ripening, with red ripe fruits harboring higher diversity and enrichment of gram-positive taxa, including *Bacillus*, *Clostridium*, *Enterococcus*, *Lactiplantibacillus*, *Litchfieldia*, and *Pediococcus*, whereas *Pseudomonas* was enriched in the mature green stage. Correlation analysis revealed a strong association between specific bacterial taxa and ripening-related metabolites, suggesting a link between microbial succession and metabolic remodeling. Together, these findings demonstrate that tomato fruit ripening involves tightly coupled metabolic and microbial dynamics, providing new insights into fruit quality formation and postharvest ecology for sustainable agriculture.

## 1 Introduction

Tomato (Solanum lycopersicum) is a widely cultivated industrial crop across the globe because of its high economic value. The total global production of tomatoes reached 192 million metric tons in 2023, with major contributions from China and the United States, according to the Food and Agriculture Organization. Cherry tomato (*S. lycopersicum* var. *cerasiforme*), a subspecies of tomato, is also widely cultivated in China and is valued for its intense flavor (Pan et al., 2023). The fruits of cherry tomatoes are typically smaller than those of ‘big fruit’ tomatoes and contain a high abundance of organic acids, sugars, and volatile organic compounds (Pan et al., 2023). Importantly, fruit size and color are intriguing visual characteristics that determine consumers’ purchase decisions (Jürkenbeck et al., 2020; Oltman et al., 2014). Tomato fruits are mainly consumed at their maximum organoleptic quality, which occurs when they reach the full red color stage but before they become overly soft (Felföldi et al., 2022).

The ripening of tomato fruit is a multifaceted process that can be categorized into six stages: green, breaker, turning, pink, light red, and fully red, which can be influenced by host genetics, environmental factors, and host-microbe interactions (Choi and Park, 2023; Rodríguez et al., 2021; Silva et al., 2021). Plant hormones and transcriptional regulators, along with other molecular mechanisms, coordinate the processes of fruit development and ripening (Wang and de Maagd, 2025). It was postulated that the accumulation of ethylene and carotenoids (lycopene and β-carotene) is involved in causing changes in tomato phenotype and color (Alexander and Grierson, 2002; Giovannoni, 2004). Importantly, these ripening stages are accompanied by distinct physiological and biochemical changes that affect tomato fruit flavor (Klee and Tieman, 2018; Shinozaki et al., 2018). For instance, the tomato fruits from mature green to red stage undergo dramatic changes in sugars, lycopene, organic acids, and phenolic compounds content (Helyes et al., 2006; Tigist et al., 2013). Sugars such as fructose and glucose, along with acids like malic and citric acid, have been linked to the sweet-sour taste in fresh tomatoes (Tieman et al., 2012). In addition, sugars are crucial for tomato fruit size, as they promote cell expansion and are crucial to the organoleptic properties, quality, and yield of the fruit (Quinet et al., 2019; Kanayama, 2017). Changes in the cell wall have been linked to alteration in the softness and juiciness of tomato fruits (Brummell, 2006).

In parallel with metabolic changes, the fruit-associated microbiota also undergoes dynamic shift in structure and composition during ripening process (Nugrahapraja et al., 2024). Fruits are colonized by diversity of beneficial and pathogenic microbiota (Hussain et al., 2026; Hussain et al., 2016; Hamid et al., 2014). Microbial communities associated with fruits, both as an epiphytes and endophyte, influence host secondary metabolites composition linked to flavor, and also play critical role in plant’s resistance to postharvest diseases and insect pests (Wu et al., 2019; Droby and Wisniewski, 2018). For example, in recent study, the mulberry fruits microbiota during ripening process have shown a positive correlation with esters, which contribute to the development of floral and fruity flavors (Bian et al., 2024). This microbial-induced alteration in fruit flavor has also been shown for strawberry fruit and Basmati rice due to activity of methylotrophic bacteria and rhizobacteria, respectively (Verginer et al., 2010; Deshmukh et al., 2016). In tomato, the fruit-associated bacterial microbiota have been linked to aroma-relevant volatile and sugars (fructose and glucose) in ripe fruits (Rodríguez et al., 2021). Beside this, the bacterial inoculation in soil have also been linked to tomato fruit flavor and nutrition quality by increasing the contents of sugars, fatty acids, and amino acids (Fu et al., 2025). Interestingly, the bacterial microbiota have also been associated with delaying in ripening process in chitosan-treated banana fruits, providing a possible approach to minimize postharvest losses and enhance the quality of banana fruit (Nugrahapraja et al., 2024). However, there is lack of studies on tomato that explore metabolic and microbial dynamics across different ripening stages, which may provide unique metabolic or microbial signatures associated with fruit ripening and quality formation.

In this study, we selected cherry tomatoes for their intense flavor and limited research to investigate how shifts in metabolic profiles correlate with fruit-associated bacterial communities during different stages of the ripening process. To achieve this, we sampled cherry tomato fruits at the mature green, pink, and red ripe stages for untargeted liquid chromatography-mass spectrometry (LC-MS) metabolomics and bacterial community analysis using high-throughput amplicon sequencing. The specific objectives of the study were to (a) characterize metabolic trajectories across three different stages of tomato ripening to identify stage-specific metabolites, (b) decipher the tomato fruit-associated microbiome to monitor bacterial microbiota dynamics as ripening proceeds, and (c) determine whether there are correlations between specific metabolic patterns and microbial taxa, which may provide evidence of metabolite-microbe interactions influencing tomato ripening. The differences in fruit metabolites and bacterial microbiota across ripening stages would have important implications for cherry tomato quality, flavor formation, and postharvest management.

## 2 Materials and methods

### 2.1 Plant materials

Cherry tomatoes (*Solanum lycopersicum* var. *cerasiforme* cv. Yu-nu) were cultivated in Yongle village, Youjiang District (23.9022° N, 106.6184° E), Baise city, China, during the spring of 2025. The cultivation was carried out in natural soil under field condition. Composite microbial fertilizer composed of effective viable bacteria, N+P_2_O_5_+K_2_O, and organic matter was applied to provide nutrient supply to the plants. In addition, plants were grown exclusively under natural sunlight. Fruit samples were collected at three defined developmental stages: mature green (GT; fully sized, green fruit), pink (PT; 50% pink color), and red ripe (RT; fully red) (**Supplementary Figure 1**). Immediately following harvest, the fruits were flash-frozen in liquid nitrogen to preserve their physiological state. All experimental procedures were performed using four biological replicates for each ripening stage.

### 2.2 Determination of sugar, lycopene and vitamin C content in tomato fruits

The soluble sugar concentration was quantified in cherry tomato fruits using the 3,5-dinitrosalicylic acid (DNS) method, as described by Wang et al. (2021). Briefly, the sample aliquot and DNS reagent were mixed in a 1:1 ratio. Next, the resulting mixture was incubated for 10 min in hot boiling water to form a red-brown color, then allowed to cool and further diluted with distilled water. A spectrophotometer was used to determine the absorbance of the final solution at 540 nm. A glucose solution with a known volume was used to generate a standard curve, and the soluble sugar content in each sample was calculated as grams (g) of glucose equivalent per kilogram (kg) of fresh weight.

A mixture of acetone/hexane (4:6 v/v) was used to extract lycopene from cherry tomato fruit samples as described by Nagata and Yamashita (1992). The suspension was centrifuged at 10,000 × g for 15 min, and the absorbance of the resulting supernatant was measured at 472 nm using a spectrophotometer. The extinction coefficient for lycopene in hexane (E1%1cm = 3450) was used to calculate the lycopene content, and reported as grams per kilogram of fresh weight.

Vitamin C content in cherry tomato samples was determined using the 2,6-dichloroindophenol (DCPIP) titrimetric as previously outlined by Arya et al. (2000)The sulfuric acid with the concentration of 3% was used for sample extraction. A measured volume of the extract was titrated with a standardized DCPIP solution until a faint pink color remained for at least 15 s. The vitamin C concentration was then calculated according to the DCPIP solution volume used, and the results were reported asascorbic acid in micrograms per gram of fresh weight.

### 2.3 Metabolomics analysis of tomato fruits

For metabolome analysis, 100 mg of tissue from each cherry tomato fruit was subsampled. The remaining fruit tissue was retained for subsequent DNA extraction and microbiome analysis (section 2.4). To profile metabolites from cherry tomato fruits using untargeted liquid chromatography-mass spectrometry (LC-MS), 100 mg of fruit was transferred into a 2 mL centrifuge tube containing a 6 mm grinding bead. Subsequently, 800 μL of extraction solution (methanol:water, 4:1 v:v) supplemented with internal standards (0.02 mg/mL L-2-chlorophenylalanine) was added to the sample. The samples were ground for 6 min at -10 °C using a Wonbio-96c tissue grinder (Shanghai Wanbo Biotechnology Co., LTD), followed by ultrasonic extraction at 5 °C for 30 min. Samples were first cooled at -20 °C for 30 min, then centrifuged at 13000 g for 15 min at 4 °C, and the resulting supernatant was placed into an injection vial for subsequent LC-MS/MS analysis.

LC-MS/MS analysis was carried out on a Thermo UHPLC-Q Exactive system (Majorbio Bio-Pharm Technology Co. Ltd. Shanghai, China) using an ACQUITY BEH Amide column (100 mm × 2.1 mm i.d., 1.7 μm; Waters, USA). The column temperature was maintained at 40 °C, with a flow rate of 0.40 mL/min and an injection volume of 3 μL. Mass spectrometric analysis was conducted using an electrospray ionization (ESI) source in both positive and negative ionization modes. The instrument was operated under the following optimized conditions: source temperature of 400 °C; sheath gas flow rate of 40 arb; auxiliary gas flow rate of 10 arb; and ion-spray voltages of -2800V for negative mode and 3500V for positive mode. The MS/MS normalized collision energy was set to 20-40-60V. The full MS resolution was 70000, and the MS/MS resolution was 17,500. Data acquisition was performed using the data dependent acquisition (DDA) mode, with detection over a mass range of 70-1050 m/z.

LC-MS raw data preprocessing was conducted using Progenesis QI (Waters Corporation, Milford, USA). Internal standard peaks and any false positives were removed, the data were cleaned, redundant peaks were removed, and metabolites were identified using the plant-specific metabolite database (MJDBPM) from Majorbio Biotechnology Co. Ltd. (Shanghai, China). The processed data were uploaded to the Majorbio Cloud platform for further analysis. The data matrix was pre-processed, retaining atleast 80% of detected metabolic features, with missing values filled using the minimum value, and normalization was performed using sum normalization. Variables with an RSD > 30% in QC samples were excluded, and log_10_ transformation was applied. The final data matrix was analyzed using Partial Least Squares Discriminant Analysis (PLS-DA) through the R-package “ropls” v1.6.2. Metabolites with VIP > 1 and p < 0.05 were considered significantly different and are shown in the volcano plot. Differential metabolites were mapped to biochemical pathways using KEGG, and enrichment analysis was performed to identify the most relevant biological pathways using the Python package “scipy.stats”.

### 2.4 DNA extraction, amplicon sequencing and bioinformatics analysis

The same individual fruits used for metabolomics (section 2.3) were used for bacterial community analysis. After subsampling 100 mg for matabolomics, the remaining tissue was processed for DNA extraction. Genomic DNA was isolated from tomato fruits by grinding them into a fine powder using liquid nitrogen in a mortar and pestle, thereby including both epiphytic and endophytic bacterial microbiota of the fruit. DNA extraction was conducted using the FastDNA Spin Kit for Soil, following the manufacturer’s instructions (MP Biomedical, Santa Ana, USA). The DNA concentrations of each sample were measured using a Qubit fluorometer (Thermo Fisher Scientific Inc., Waltham, MA, USA). The extracted DNA was stored at -80 °C prior to microbial community analysis.

To profile the bacterial community in cherry tomato fruits, the V5-V7 region of the 16S rRNA gene was targeted. PCR amplification was carried out using the primers 799F (5’-AACMGGATTAGATACCCKG-3’) and 1193R (5’-ACGTCATCCCCACCTTCC-3’) (Bodenhausen et al., 2013), under the following conditions: 95 °C for 3 min, followed by 35 amplification cycles at 95 °C for 30 s, 55 °C for 30 s, and 72 °C for 45 s, with a final elongation step at 72 °C for 10 min. The amplified products were purified, pooled, and sequenced using an Illumina PE250 platform (Majorbio, Shanghai, China).

The raw FASTQ files were first demultiplexed using a Perl script and then subjected to quality filtering with fastp (v0.19.6). Sequences were merged using FLASH (v1.2.7) under the following conditions: (i) sequences with average quality score of <20 in a 10 bp sliding window were truncated at the corresponding position. Sequences shorter than 100 bp after trimming or those containing ambiguous bases were removed; (ii) paired-end reads were merged with a minimum overlap of 10 bp and a maximum mismatch ratio of 0.2 in the overlapping region. Any read pairs that could not be merged were discarded; (iii) quality sequences were assigned to each sample based on barcode, primer matching allowing for up to 2 base mismatches in the primer region, and adjusting for sequence orientation.

After quality control, denoising was performed using the DADA2 algorithm within the QIIME2 (v2024) pipeline, with the recommended parameters, to achieve single-nucleotide resolution. After this process, the resulting sequences were referred to as ampliconsequence variants (ASVs). The representative reads were taxonomically classified against the SILVA 16S rRNA database (v138.2) for bacteria (Quast et al., 2013), using the Naive Bayes consensus taxonomy classifier. To minimize the effect of sequencing depth differences, all cherry tomato samples were rarefied to 41688 sequences per sample for bacteria before downstream analysis.

### 2.5 Statistical analysis

Statistical analysis of the bacterial communities associated with different ripening stages of cherry tomato fruits were conducted using R software (v4.4.2). Sequences identified as plant mitochondria and chloroplasts were excluded prior to data analysis. To assess the alpha diversity of bacterial communities, the Shannon diversity index and Simpson index were computed using the R package ‘vegan’. Treatment means for the various ripening stages were compared using analysis of variance (ANOVA), followed by LSD tests. Beta diversity of bacterial communities was performed using Bray-Curtis distance based on the relative abundance (RAs) of ASVs using the R package ‘phyloseq’. Principal coordinate analysis (PCoA) plots were generated from the Bray-Curtis distance matrix. Venn diagram was generated to mark unique and shared ASVs using the R package VennDiagram. To assess the significance of community composition differences across ripening stages, bar graphs were generated using the R package ‘ggplot2’ to visualize the average RAs of bacterial phyla across different ripening stages. Core bacterial taxa were defined as ASVs identified at the genus level that appeared in minimum 50% of samples from each ripening stage and had a minimum relative abundance of 0.01%, as described by Abera et al. (2022). Enriched bacterial ASVs at the green, pink, and red stages were visualized using a volcano plot. The correlations between bacterial microbiota and metabolites were performed using the Spearman’s rank correlation using the R package ggcorrplot.

## 3 Results

### 3.1 Effect of ripening treatments on cherry tomato quality

**Figure 1:**
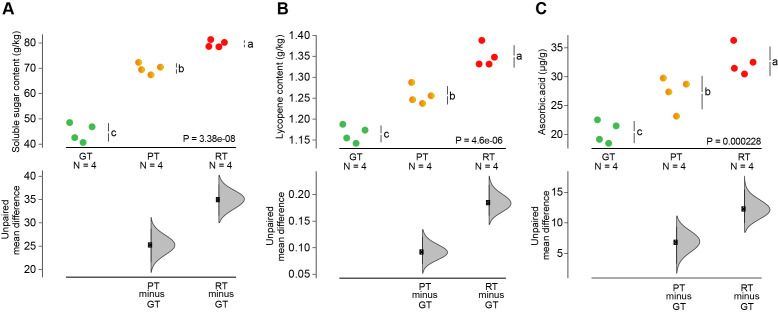
Gardner-Altman estimation plot show the difference for **(A)** soluble sugar content, **(B)** lycopene content, and **(C)** ascorbic acid content across three tomato ripening stages: mature green (GT), pink (PT), and red ripe (RT). The black vertical line represents the difference in mean values between the two group (PT or RT compared to GT), accompanied by a 95% confidence interval. The shaded area within the whole gray curve indicates the distribution of errors. Statistical significance between three ripening stages is indicated by different letters based on LSD test (P < 0.05).

The different ripening stages of cherry tomatoes showed significant variations in fruit quality parameters. Specifically, the tomatoes showed substantially improved fruit quality at the red ripening stage, as indicated by the average contents of soluble sugar, lycopene, and ascorbic acid (**Figure 1**). The soluble sugar content was significantly shifted from green to red cherry tomatoes, with RT exhibiting the highest soluble sugar content, followed by PT and GT (P = 3.38e-08; **Figure 1A**). Similarly, lycopene content was highest in RT, followed by PT and GT (P = 4.66e-06; **Figure 1B**). For ascorbic acid (vitamin C) content, RT displayed the highest concentration, which significantly differed from PT and GT with lower values (P = 2.28 e-04; **Figure 1C**).

### 3.2 Cherry tomato fruit metabolomic changes across ripening stages

**Figure 2:**
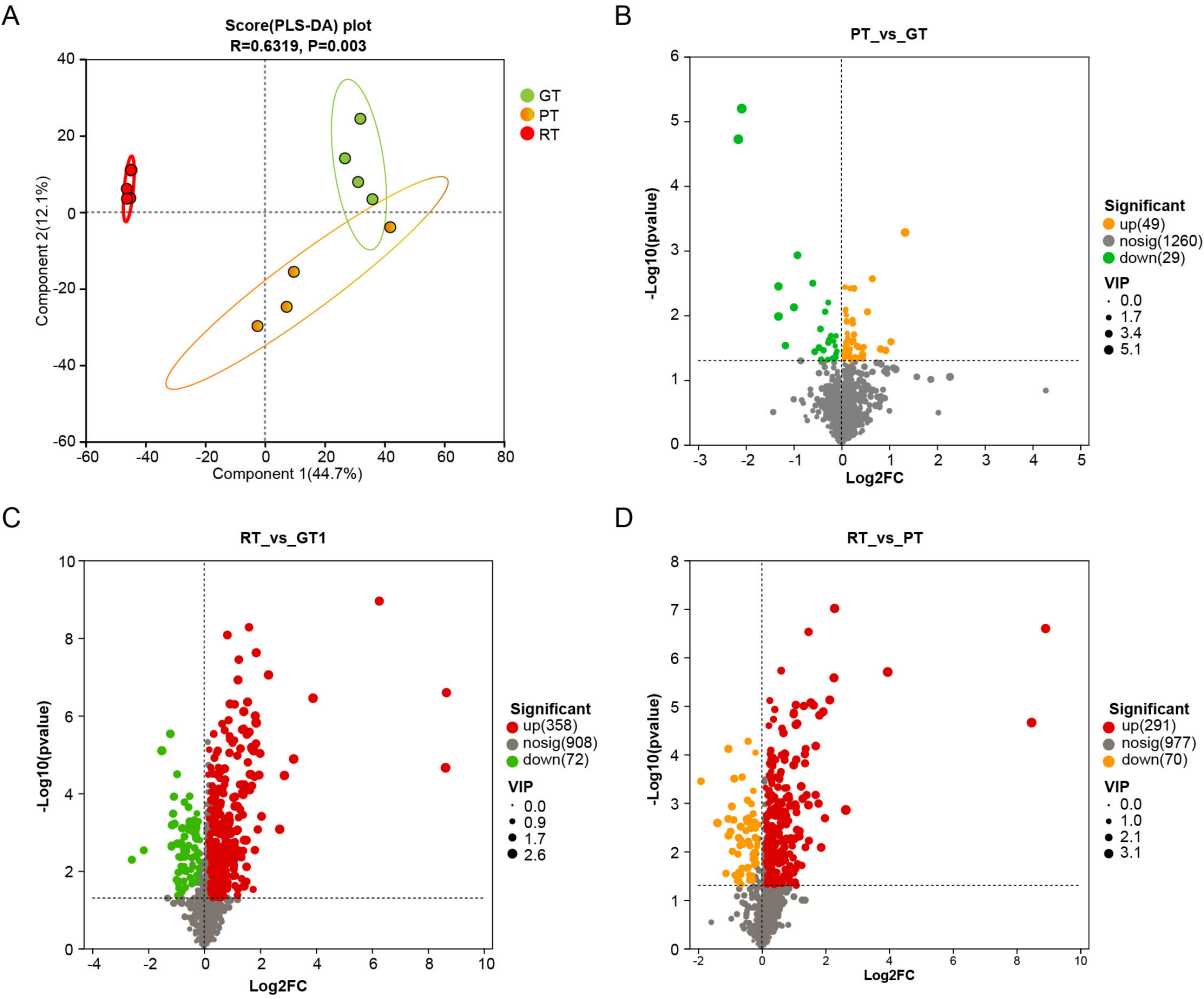
Metabolic profiling analysis in cherry tomato. **(A)** PLS-DA plot showing separation between three ripening stages: mature green (GT), pink (PT), and red ripe (RT). **(B)** Volcano plot comparing PT with GT, highlighting significantly more abundant (orange), less abundant (green), and non-significant (gray) metabolites. **(C)** Volcano plot comparing RT with GT, highlighting significantly more abundant (red), less abundant (green), and non-significant (gray) metabolites. **(D)** Volcano plot comparing RT with PT, highlighting significantly more abundant (red), less abundant (orange), and non-significant (gray) metabolites. The VIP values are indicated by dot size.

The PLS-DA analysis showed a clear separation between the early (GT) and late ripening stages (RT) of tomato fruits (**Figure 2A**). In contrast, GT and PT clustered more closely, indicating partial differentiation of metabolic profiles between adjacent stages. The component 1 explained 44.7% of the variance, and component 2 accounted for 12.1% of the variance. Overall, the clustering pattern suggested that ripening stage influences metabolic profiles (**Figure 2A**).

The volcano plot revealed significant changes in the metabolite numbers across the three ripening stages. A comparison of PT with GT showed that 49 metabolites were significantly more abundant (orange circles), and 29 metabolites were significantly less abundant (green circles) in PT than in GT. The VIP values indicated that these metabolites substantially contributed to the differences between the two ripening stages. In the comparison between RT and GT, a pronounced shift was observed in the number of differential metabolites. A total of 358 metabolites were significantly more abundant (red), whereas 72 metabolites were significantly less abundant (green) in RT than in GT. Finally, a notable difference was observed between the RT and PT stage. A total of 291 metabolites were significantly more abundant (red) and 70 metabolites were less abundant (orange) in the RT than PT. This data suggests that the transition from the green to pink to red stages in cherry tomatoes involves significant metabolic changes.

### 3.3 Metabolite composition and pathway enrichment analysis of tomato fruits at different ripening stages

The metabolite analysis of cherry tomatoes at different ripening stages revealed the distribution of primary metabolites like lipids (38.63%), amino acids and derivatives (28.36%), carbohydrate and derivatives (23.47%), nucleotide and derivatives (5.56%), and vitamins (0.98%) (**Supplementary Figure 2**). In the secondary metabolite category, terpenoids (26.81%) and flavonoids (21.91%) were the most dominant, followed by steroids and steroid derivatives (13.75%), phenolic acid and derivatives (13.05%), and organic acid and derivatives (7.23%) (**Supplementary Figure 3**). Other secondary metabolites, including coumarin and derivatives (4.66%), indoles and derivatives (3.73%), alkaloids and derivatives (3.5%), lignans and derivatives (2.56%), quinones (2.1%), and tannins (0.7%) were present in smaller amounts (**Supplementary Figure 3**).

**Figure 3:**
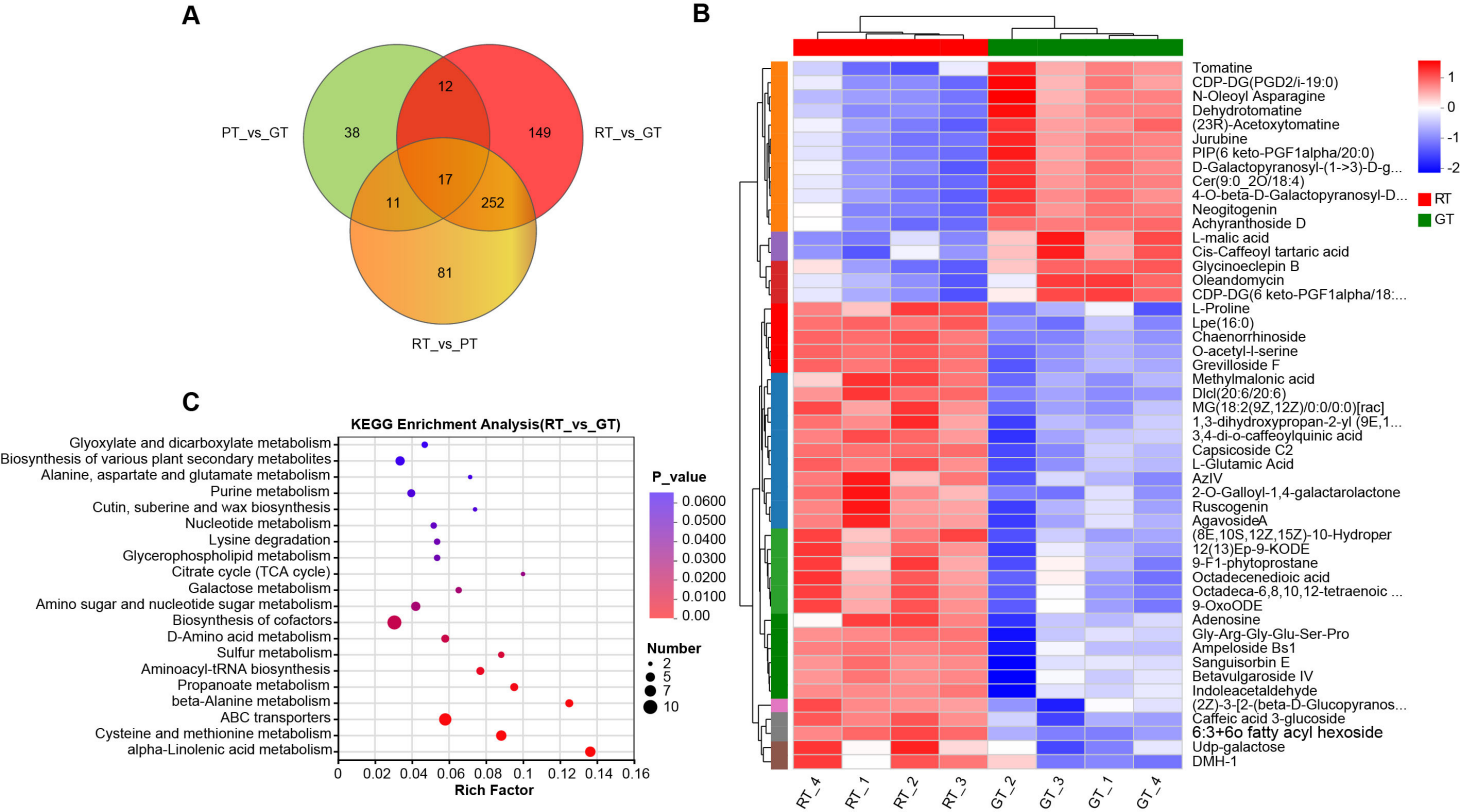
Metabolites profiling of cherry tomatoes at different ripening stages. **(A)** Venn diagram showing the overlap of differentially expressed metabolites between RT vs GT, RT vs PT, and PT vs GT. **(B)** Heatmap of top 50 significantly differential metabolites in RT and GT samples, where red represents higher expression and blue represents lower expression. **(C)** KEGG enrichment analysis highlighting the significantly enriched metabolic pathways in RT compared to GT.

The pair-wise comparison of significantly different metabolites at different ripening stages showed the number of unique and shared metabolites. A total of 38, 81, and 149 unique metabolites were identified in the PT vs GT, RT vs PT, and RT vs GT groups, respectively. Importantly, 17 metabolites were consistently associated with different ripening stages in pair-wise group comparisons between GT, PT and RT (**Figure 3A**).

A comparison of the red and green stages identified key abundant metabolites that were differentially present in both stages. Metabolites such as tomatine, dehydrotomatine N-oleoyl asparagine, jurubine, neogitogenin, L-malic acid, cis-caffeoyl tartaric acid, glycinoeclepin B, and oleandomycin were significantly more abundant in GT (**Figure 3B**). In contrast, metabolites such as L-proline, chaenorrhinoside, grevilloside F, methylmalonic acid, L-glutamic acid, ruscogenin, agavoside A, octadecenedioic acid, and UDP-galactose were significantly more abundant in RT than in GT (**Figure 3B**). Interestingly, most GT-enriched metabolites were more abundant in PT than in RT (**Supplementary Figure 4A**). Similarly, some of the differentially abundant metabolites enriched in RT than GT were also identified to be more abundant in PT than in GT (**Supplementary Figure 5A**). A comparison of PT with GT revealed unique changes in the metabolite composition. In GT, metabolites such as phenacylamine, traumatic acid, azelaic acid, and carboprost were significantly more abundant (**Supplementary Figure 5A**). Metabolites such as enoxacin, cinnamoside, dictyoquinazol B, gamma-aminobutyric acid, L-glutamic acid, farnesyl acetone, adenosine monophosphate, adenosine 3’-monophosphate, and tyramine were enriched in the PT as compared to the GT (**Supplementary Figure 5A**).

The KEGG enrichment analysis comparing RT with GT showed several enriched metabolic pathways. Notably, alpha-linolenic acid metabolism, cysteine and methionine metabolism, ABC transporters, beta-alanine metabolism, propanoate metabolism, aminoacyl-tRNA biosynthesis, sulfur metabolism, D-amino acid metabolism, biosynthesis of cofactors, galactose metabolism, citrate cycle (TCA cycle), and amino sugar and nucleotide sugar metabolism showed the highest rich factor and the lowest p-value <0.05 (**Figure 3C**). Several of these metabolic pathways were also enriched in RT compared to the PT (**Supplementary Figure 4B**). A comparison of PT and GT revealed enrichment of metabolic pathways such as alanine, aspartate and glutamate metabolism, zeatin biosynthesis, alpha-linolenic acid metabolism, butanoate metabolism, arginine and proline metabolism, sulfur relay system, and purine metabolism (**Supplementary Figure 5B**).

### 3.4 Bacterial community diversity in cherry tomato across ripening stages

**Figure 4:**
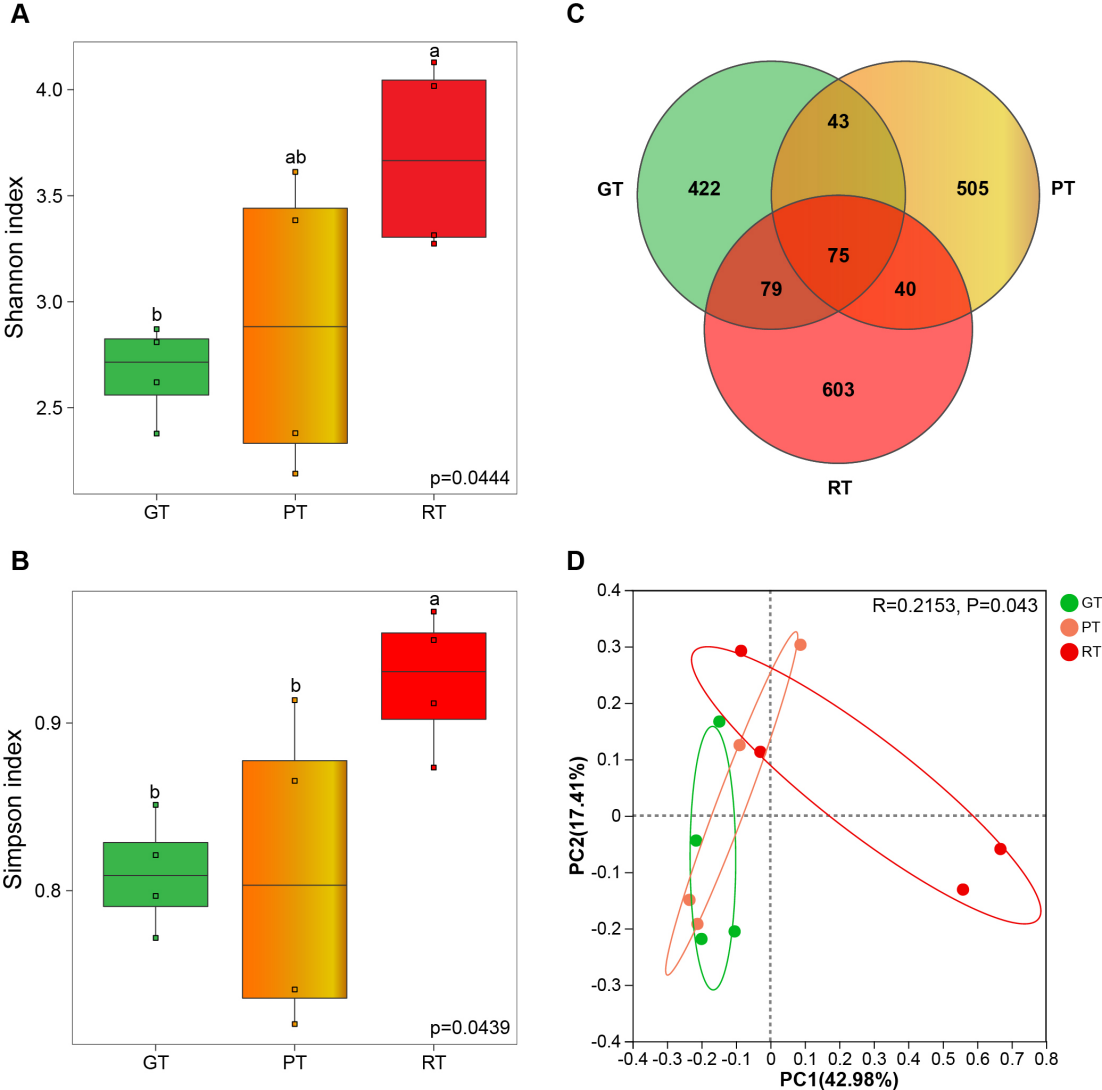
Bacterial community diversity of cherry tomato fruit at different ripening stages. Boxplot of the Shannon index **(A)** and Simpson index **(B)**, indicating differences in alpha diversity between GT, PT and RT. Different letters appearing above the boxes indicate significant differences between groups based on the LSD test at p < 0.05. **(C)** Venn diagram of bacterial ASVs across the three ripening stages. **(D)** PCoA analysis of bacterial community inhabiting the cherry tomato fruit at three different ripening stages.

Bacterial community analysis of cherry tomato fruits at different ripening stages revealed significant differences in diversity metrics. The Shannon index values were significantly higher in RT than in GT, whereas the diversity value of PT was not significantly different from that of GT and RT (**Figure 4A**). The Simpson index also displayed similar trends, but the RT values were significantly higher than the PT and GT (**Figure 4B**). The Venn diagram indicates the overlap of bacterial communities between the different ripening stages, showing that RT had a higher number of unique ASVs, followed by PT and GT, complementing the results of the alpha diversity analysis. A total of 75 ASVs were commonly shared between the three ripening stages (**Figure 4C**). PCoA analysis of the bacterial community showed a trend toward separation based on ripening stage, although with substantial overlap among groups (R = 0.2153, P = 0.043). RT displayed the greatest compositional difference compared to GT and PT. The axis 1 explained a total of 42.98% variation and the axis 2 explained 17.4% variation in the bacterial communities (**Figure 4D**).

### 3.5 Bacterial community composition in cherry tomato across ripening stages

**Figure 5:**
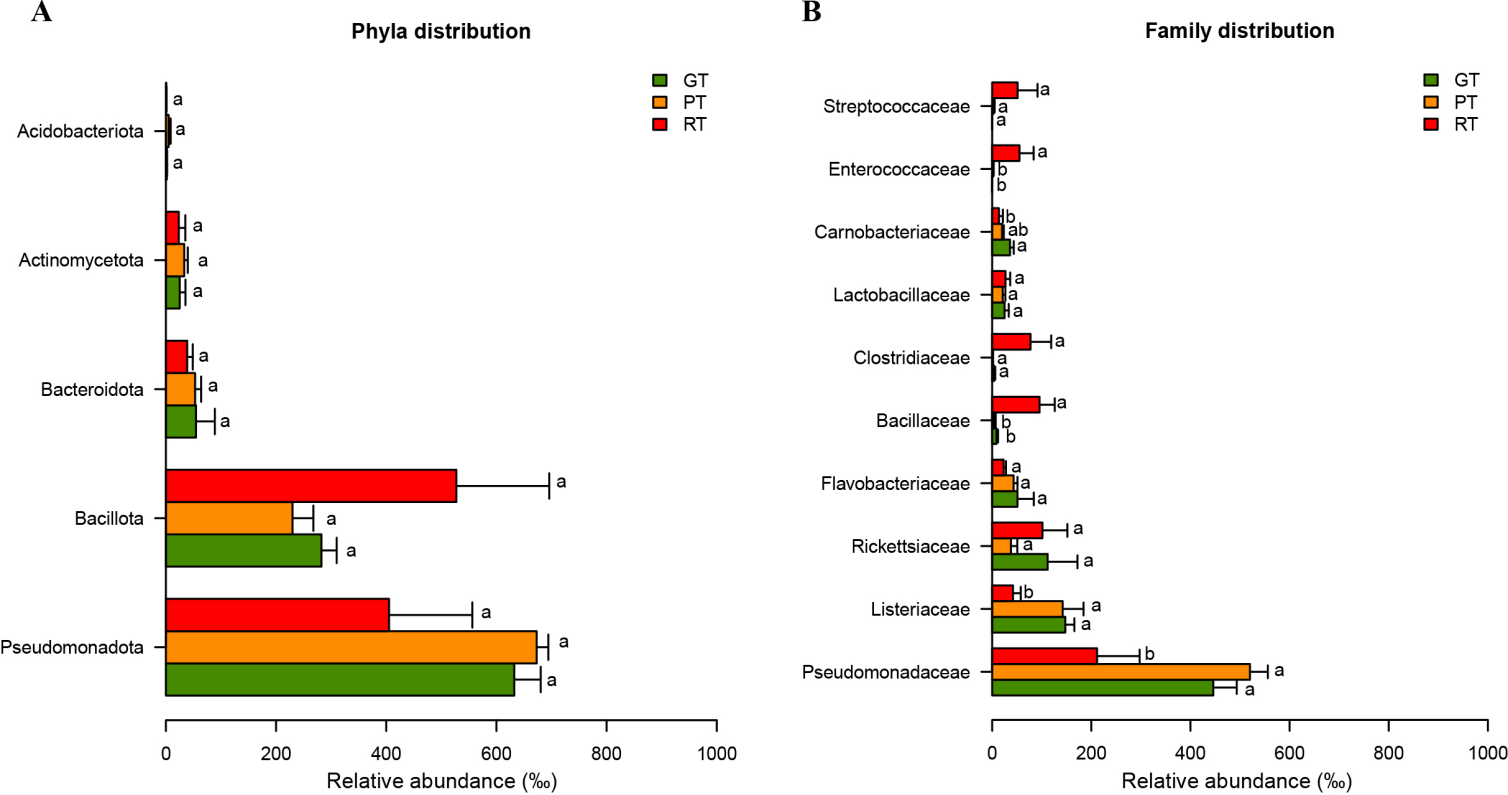
Bacterial community composition in cherry tomato fruits at different ripening stages. **(A)** The relative abundance of bacterial phyla in tomato fruits at GT, PT and RT stages. **(B)** The relative abundance of bacterial families in tomato fruits at GT, PT and RT stages. Different letters appearing on the bars indicate significant differences between groups based on the LSD test at p < 0.05.

The bacterial community composition at the phylum level showed the dominance of Pseudomonadota, Bacillota, Bacteroidota, Actinomycetota, and Acidobacteria on the cherry tomato fruits. The relative abundances of these phyla were not significantly different across the three ripening stages (**Figure 5A**).

However, we observed significant differences in the fruit-associated bacterial community composition at the family level. Notably, Pseudomonadaceae and Listeriaceae showed significantly higher abundance in GT and PT than in RT (**Figure 5B**). The relative abundances of Bacillaceae, Clostridiaceae, Enterococcaceae, and Streptococcaceae were significantly higher in the RT than in the GT and PT (**Figure 5B**). The family Carnobacteriaceae also exhibited significant variation across the three ripening stages, with a high relative abundance in GT, followed by PT and RT.

### 3.6 Core and enriched bacterial microbiota in cherry tomato

**Figure 6:**
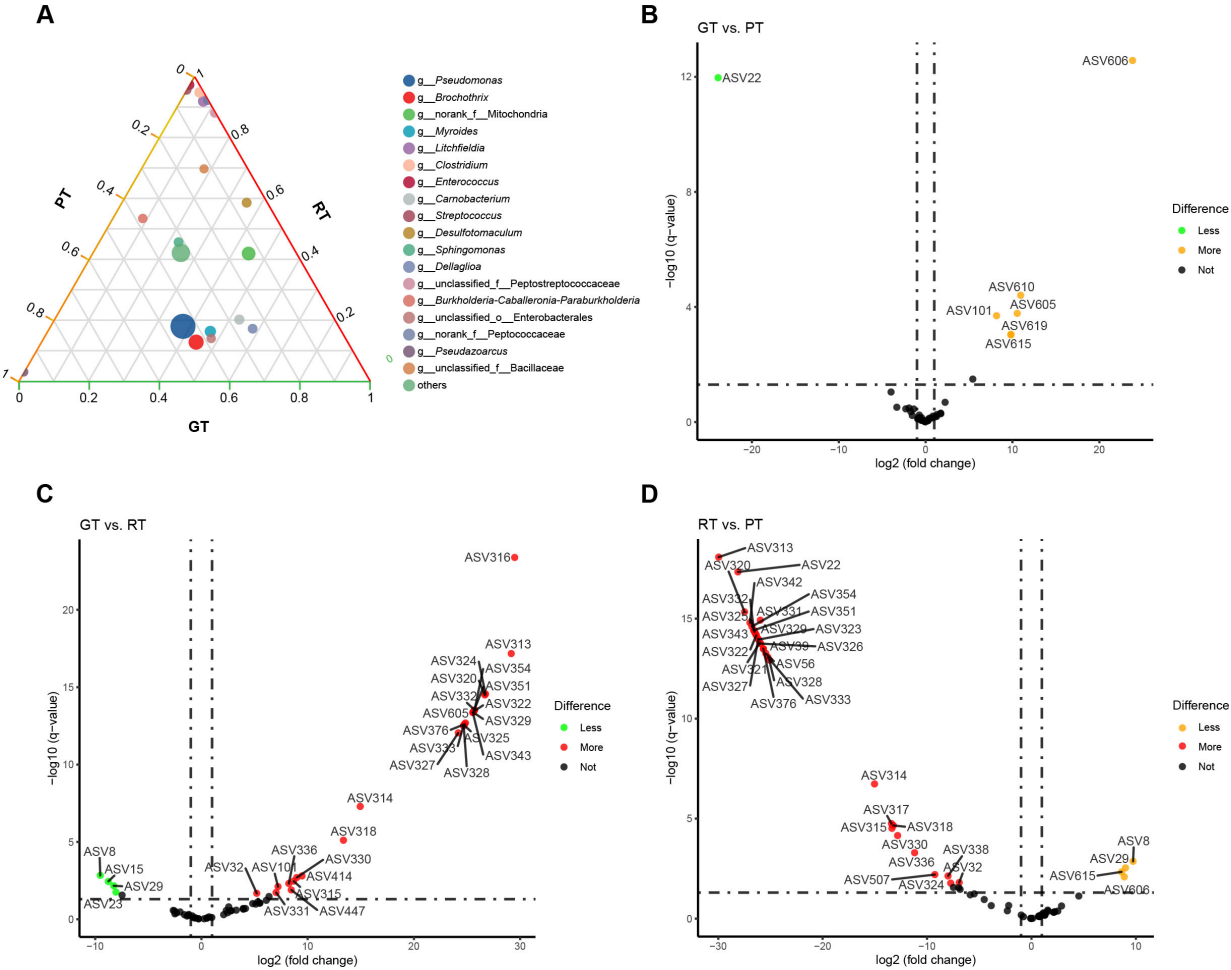
Bacterial enrichment in cherry tomato across three ripening stages. **(A)** Ternary plot showing the relative abundance of bacterial taxa at each ripening stage. The size of circle represents their relative abundance and the color of each circle indicates the bacterial genera it belongs to. **(B-D)** Volcano plots showing significantly enriched ASVs between different ripening stages: **(B)** GT vs PT, **(C)** GT vs RT, and **(D)** RT vs PT. Dots represent individual ASVs, with different color indicating the significance of differences in each stage (green: more abundant in GT stage, orange: more abundant in PT stage, red: more abundant in RT stage, and black: no significant difference).

The ternary plot showed the relative abundance of dominant bacterial genera at different ripening stages of cherry tomato fruit. The bacterial genera composition altered significantly across the GT, PT, and RT stages. The relative abundances of *Pseudomonas*, *Brochothrix*, *Myroides*, *Carnobacterium* and *Dellaglioa* were higher in GT and PT than in RT (**Figure 6A**). The genera *Litchfieldia*, *Clostridium*, *Enterococcus*, *Streptococcus*, *Sphingomonas*, *Burkholdera-Caballeronia-Paraburkholderia* were more abundant in the RT than in the GT and PT (**Figure 6A**), indicating a shift in the bacterial community at the genus level as the cherry tomato ripened. Importantly, our analysis of the core bacterial genera across the three ripening stages identified *Pseudomonas*, *Brochothrix*, *Myroides*, *Carnobacterium*, *Desulfotomaculum*, *Sphingomonas*, *Dellaglioa*, *Burkholderia-Caballeronia-Paraburkholderia*, *Vagococcus*, *Methylobacterium*, *Corynebacterium*, *Vibrio*, *Shewanella*, *Acinetobacter*, and *Cutibacterium* as the core taxa in cherry tomatoes (**Supplementary Figure 6**).

The volcano plot further showed a significant difference in the bacterial community at the ASV level. A comparison of GT and PT revealed fewer differentially abundant ASVs, with ASV22 belonging to the bacterial order Peptostreptococcales enriched in GT, and ASV101 (g:*Pseudomonas*), ASV605 (g:*Paracoccus*), ASV606 (g:*Pseudazoarcus*), ASV610 (g:*Levilactobacillus*), ASV615 (g:*Parazoarcus*), and ASV619 (g:*Burkholderia-Caballeronia-Paraburkholderia*) enriched in the PT stage (**Figure 6B**; **Supplementary Table S1**). A strong effect of ripening on the bacterial community at the ASV level was observed between RT and GT or PT. A comparison of RT with GT showed 26 enriched ASVs in RT, whereas GT had only 4 enriched ASVs (**Figure 6C**;**Table S1**). The enriched ASVs in RT relative to GT belonged to the bacterial genera *Streptococcus*, *Enterococcus*, *Fonticella*, *Clostridium*, *Escherichia-Shigella*, *Lactiplantibacillus*, *Bacillus*, *Paracoccus*, *Pediococcus*, *Litchfieldia*, *Flavobacterium*, and *Pseudomonas* (**Figure 6C**; **Table S1**). ASVs belonging to *Pseudomonas* and unclassified taxa were also enriched in the GT as compared to the RT (**Figure 6C**; **Supplementary Table S1**). Interestingly, we observed a higher number of ASVs enriched in RT (31 ASVs) than in PT (4 ASVs; **Figure 6D**; **Table S1**). The RT stage was enriched by ASVs belonging to *Enterococcus*, *Romboutsia*, *Bacillus*, *Clostridium*, *Escherichia-Shigella*, *Fonticella*, *Desulfonispora*, *Pediococcus*, *Litchfieldia*, and *Lactiplantibacillus* (**Figure 6D**; **Table S1**). In contrast, the PT stage was enriched by *Pseudomonas*, *Parazoarcus* and *Pseudazoarcus* compared to the RT stage (**Figure 6D**).

### 3.7 Correlation analysis of enriched bacterial taxa with core metabolites in cherry tomato fruits

**Figure 7:**
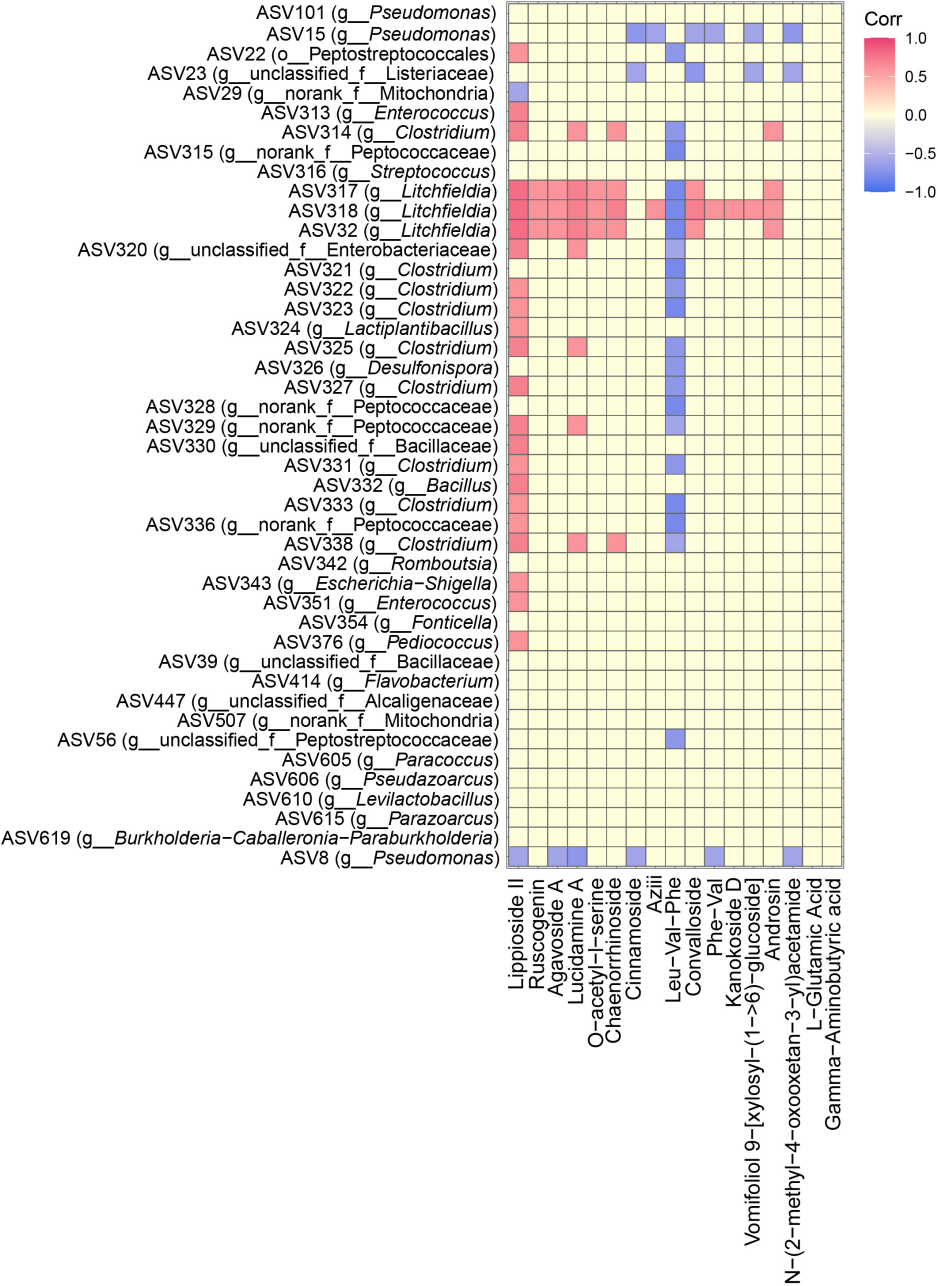
Correlation heatmap showing the relationships between the enriched ASVs and metabolites in cherry tomato fruits. Red color boxes indicate positive correlation, while blue boxes indicate negative correlations. The ASVs are labeled by their respective genera or family or order based on taxonomic annotation.

The correlation heatmap revealed the positive and negative association between several of enriched ASVs across three ripening stages and the 17 core metabolites that were consistently associated with different ripening stages in pair-wise group comparison between GT, PT and RT (**Figures 3A**, **7**). The ASVs, which belonged to diverse genera such as *Pseudomonas*, *Enterococcus*, *Clostridium*, *Litchfieldia*, *Lactiplantibacillus*, *Desulfonispora*, *Bacillus*, *Escherichia-Shigella*, and *Pediococcus* displayed varying degree of correlation with distinct metabolites (**Figure 7**). *Pseudomonas* associated ASVs showed significantly negative correlation with numbers of metabolites, including lippioside II, Agavoside A, Lucidamine A, Cinnamoside, Aziii, Convalloside, Phe-Val, Vomifoliol 9-[xylosyl-(1->6)-glucoside], and N-(2-methyl-4-oxooxetan-3-yl)acetamide (**Figure 7**). Specifically, the metabolite lippioside II was positively correlated with the most of the bacterial ASVs (linked to *Enterococcus*, *Clostridium*, *Litchfieldia*, *Lactiplantibacillus*, *Bacillus*, *Escherichia-Shigella*, and *Pediococcus*), and the metabolite Leu-Val-Phe was negatively correlated with most of these bacterial ASVs (**Figure 7**). In contrast, the bacterial genus *Litchfieldia* was positively correlated with most of the metabolites, such as Lippioside II, Ruscogenin, Agavoside A, Lucidamine A, O-acetyl-l-serine, Chaenorrhinoside, Aziii, Convalloside, Phe-Val, Kanokoside D, Vomifoliol 9-[xylosyl-(1->6)-glucoside], and Androsin (**Figure 7**). Interestingly, most of the core genera including *Pseudomonas*, *Brochothrix*, *Carnobacterium*, *Dellaglioa*, *Vagococcus*, and *Vibrio* were negatively correlated with the core metabolites that were consistently enriched across different ripening stages in pair-wise group comparison between GT, PT and RT (**Figure 3A**, **Supplementary Figure 7**).

## 4 Discussion

The fruit ripening is a complex process influenced by multiple factors such as host genetics, changes in environmental factors, and host-microbe interactions. In the present study, we asked whether cherry tomatoes at different ripening stages differ in metabolite and bacteriome composition, and whether such changes in fruit chemistry may be linked to the fruit-associated bacterial microbiota. To this end, we observed a significant shift in biochemical, metabolic and bacterial community composition as the tomato ripened from the GT to PT, and finally to the RT stage. A significant increase in sugar, lycopene and vitamin C contents was monitored from green to red tomatoes. This observation aligns with the previous studies showed that the sugars content of tomato fruits increases as fruits proceeded from green to red ripe stage (Kaur et al., 2007). Sugars give sweet taste and are vital to cell expansion process regulating development and metabolism of tomato fruits (Kanayama, 2017). Sugars are usually formed in plant’s photosynthetic tissues, and are transferred to the fruits via long-distance transport through phloem, where they are then distributed throughout the fruit tissues via the apoplast and symplast (Reuscher et al., 2014). Beside sugars, It has also been observed in previous studies that the content of lycopene and organic acids undergo significant changes as they ripened from GT to RT stage (Helyes et al., 2006). Lycopene, a carotenoid with antioxidant properties, is highly abundant in fruit and contribute to red coloration (Ilahy et al., 2018). It is worth noting that the interplay between sugars and organic acids determine tomato fruit sweetness and sourness, which indicates the overall taste quality (Zhu et al., 2018).

Fruit aroma and flavor development are usually linked to the tomato ripening process (Helyes et al., 2006). In our study, metabolomic profiling of tomatoes at distinct ripening stages supported the hypothesis that fruits undergo dramatic metabolic reprogramming during fruit ripening and maturation. We detected high abundance of primary metabolites, such as lipids, amino acids, and carbohydrates in tomatoes. Lipid components in fruits are known precursors of aroma volatiles and flavors during tomato ripening (Baldwin et al., 2000). Amino acids are precursors of tomato aroma during fruit ripening (Rambla et al., 2013). Carbohydrate metabolism has been documented to contribute to fruit development, yield and quality (Quinet et al., 2019). However, because we did not directly measure volatile organic compounds or postharvest traits, these inferred links to aroma and flavor remain speculative and require targeted validation.

We further observed the dominance of terpenoids and flavonoids among the secondary metabolites. Terpenoids are important for different plant processes, including the regulation of plant growth (hormones gibberellins) and energy transfer (carotenoids) (Gutensohn and Dudareva, 2016). Terpenoids also play a critical role in attracting pollinators, plant-microbe interactions, and disease suppression (Boncan et al., 2020; Hussain and Heuer, 2025). In contrast, the high abundance of flavonoids in tomato fruit has a significant beneficial effect on human health (Kaakoush and Morris, 2017). Flavonoid accumulation in the pericarp gives tomato fruits a characteristic red hue, which is an important attribute that markedly influences the commercial value (Slimestad et al., 2008). Importantly, alkaloids such as tomatine and dehydrotomatine were found to be significantly more abundant in mature green tomatoes than in ripe tomatoes, which were analyzed together as tomatine in previous studies (Friedman, 2015; Tamasi et al., 2019; Ballester et al., 2016). It has been previously documented that the tomatine levels in green tomatoes were much higher than those in red tomatoes (Friedman, 2015; Tamasi et al., 2019). The level of bitter-tasting tomatine is usually high in the early stages of tomato fruit development, and decreases during ripening because of its conversion into other acetyl glucosylated forms (esculeoside A or lycoperoside G and F), which are not bitter (Tohge and Fernie, 2015; Ballester et al., 2016). In addition, tomatine has been demonstrated to be involved in biological functions associated with plant defense against pathogens and insects (Liu et al., 2023). We further observed a high abundance of organic acids such as L-malic acid and cis-caffeoyl tartaric acid, in GT relative to RT stage tomatoes.

Tomato fruit naturally accumulate organic acid (e.g., citric acid and malic acid, tartaric acid, oxalic acid, and shikimic acid) as they primarily originate from tricarboxylic acid cycle (TCA) cycle (Xin-Juan et al., 2012). Malic acid is recognized as a key indicator of fruit ripening and the content of malic acid gradually decrease during the tomato fruit ripening process (Li et al., 2023). In contrast, red ripe tomatoes were enriched in metabolites such as L-proline, L-glutamic acid, and UDP-galactose, all of which showed significantly higher abundance in RT than GT (**Figure 3B**). L-glutamate is one of the most abundant amino acids associated with the characteristic ‘‘umami’’ flavor of tomato fruits, and its concentration has been reported to increase markedly during the ripening process (Boggio et al., 2000; Pratta et al., 2004). Proline is a multifunctional amino acid involved in stress responses (Huang et al., 2025), and has been implicated in the formation of desirable taste attributes in tomato fruits through its interaction with the umami amino acids such as glutamate and bitter amino acids such as arginine (Arg), thereby contributing to overall flavor profile (Alvarez et al., 2022; Liao et al., 2022; Wang et al., 2020). The enrichment of UDP-galactose in red ripe tomato fruit is likely indicative of enhanced recycling of galactose released from cell wall polysaccharides during ripening-associated cell wall disassembly. Previous study have shown that tomato fruit ripening is accompanied by a net loss of galactosyl residues from the cell wall without major changes in the activities of enzymes involved in galactose metabolism (Kim et al., 1991), supporting the view that increased UDP-galactose abundance reflects galactose turnover rather than *de novo* synthesis of cell wall component.

As discussed above, tomato fruits undergo significant changes in primary and secondary metabolites from mature green to red ripening phase. These changes in metabolic profile of tomato could influence the composition of microbiome and susceptibility of fruits to diseases. Plant-associated microbiota play critical role in plant health and productivity (Hussain et al., 2024). A previous study on mango highlighted the changes in the dynamic of fruit stem-associated microbial communities, and specifically found the presence of multiple stem-end rot pathogens along with other taxonomically diverse microbial taxa with their abundance influenced by fruit ripening (Diskin et al., 2017). In this study, we found high diversity of bacterial community in red ripe fruits dominated by the Pseudomonadota, Bacillota and Bacteroidota. The high relative abundance of these phyla may be attributed to their ability to proliferate in a nutrient-rich (copious) environments, such as tomato fruits, which provide a wide range of carbon sources including carbohydrates, amino acids, and lipids, enabling their survival under changing conditions during fruit ripening (Peighamy-Ashnaei et al., 2007; Kazakov et al., 2009; Xia et al., 2015). Importantly, we observed a significant decline in the relative abundance of Gram-negative family Pseudomonadaceae and an increase in Gram-positive families Bacillaceae, Clostridiaceae, Enterococcaceae, and Streptococcaceae during fruit ripening. This pattern is consistent with reports from grape berry system, where Gram-negative bacterial communities decline as the season progresses toward maturity, while Gram-positive communities increase in abundance (Martins et al., 2012). This alteration in microbial abundances reflects ripening-associated changes in fruit chemistry and microhabitat structure that favors Gram-positive taxa over Gram-negative bacteria.

Our results further revealed several core bacteria genera in tomato fruits including *Pseudomonas*, *Sphingomonas* and *Methylobacterium*. Importantly, the core bacterial genera such as *Vibrio*, *Shewanella* and *Cutibacterium* are not typical tomato phyllopshere inhabitants, but their presence in our core microbiome may be explained by recent evidence showing plant-associated roles for these genera (Kulanthaivel and Rameshkumar, 2025; Manjunatha et al., 2022; Campisano et al., 2014). It has been documented that the phyllosphere of eudicot plant species is mainly enriched by these three bacterial genera *Methylobacterium*, *Pseudomonas*, *Sphingomonas* (Delmotte et al., 2009; Vorholt, 2012). Interestingly, we observed a high relative abundance of *Pseudomonas*, with two ASVs (ASV8 and ASV15) significantly enriched at GT, whereas only a single *Pseudomonas* ASV (ASV101) was enriched at RT. Janisiewicz and Buyer (2010) previously reported that *Pseudomonas* spp. abundance tend to decline in the nectarine fruit as the fruit ripen. Our result on the enrichment of multiple *Pseudomonas* ASVs at GT but only a single ASV at RT suggest strain-level niche differentiation within the genus, with GT supporting a broader range of *Pseudomonas* lineages, while RT imposes stronger selective pressure that favor only specific, adaptive strains. Members of the genus *Pseudomonas* have been reported as both beneficial (Ghazanfar et al., 2016) and pathogenic (Wilson et al., 2002) on the tomato fruit surface, however, their role is strongly strain- and context-dependent. Other bacterial ASVs enriched in RT relative to GT included *Bacillus*, *Flavobacterium*, *Lactiplantibacillus*, *Litchfieldia*, *Paracoccus*, and *Pediococcus*. *Bacillus* spp. is reported as biocontrol agent and suppresses fungal pathogens on the tomato fruit surface (Punja et al., 2016). Many *Flavobacterium* spp. are recognized as important for plant growth promotion, disease suppression, and abiotic stress tolerance (Seo et al., 2024). *Lactiplantibacillus* is recognized as potential probiotic and antifungal agents (Vasundaradevi et al., 2025). *Litchfieldia* spp. has been recently found to have plant-growth promoting properties (Wang et al., 2026). *Paracoccus* enrichment in RT suggests a potential role fruit microbiome restructuring. Previous studies have reported the involvement of *Paracoccus* in stress mitigation and modulation of microbial community structure (Soaud et al., 2011; Li et al., 2026). *Pediococcus* spp. has shown to exhibit biocontrol properties against bacterial and fungal pathogens (Nguyen et al., 2024). Correlation analysis between enriched ASVs and core metabolites indicated strong coupling between microbial succession and metabolic reprogramming during ripening. *Pseudomonas*-associated ASVs enriched at GT stage than RT showed predominantly negative correlations with metabolites, suggesting consumer-oriented ecological role characteristics of early ripening stages (Silby et al., 2011). In contrast, *Litchfieldia* displayed broad positive correlations with metabolites, implying adaptation to metabolite-rich red ripe stage environments. The identification of lippioside II as a central metabolite positively associated with diverse taxa suggests a potential role for this secondary metabolite in shaping bacterial community structure (Cordovez et al., 2019; Faust and Raes, 2012). However, these correlation-based associations are exploratory and do not imply causation; experimental validation would be required to establish a causal role. Collectively, these results suggest functional differentiation among microbial taxa during ripening, with metabolite dynamics shaping microbial turnover and niche specialization.

## 5 Conclusion

Based on the integrated results of metabolic and microbial dynamics during cherry tomato fruit ripening, our findings indicate that ripening from green to red stage is marked by extensive metabolic reprogramming, including the accumulation of sugars, amino acids, carotenoids, and secondary metabolites linked to fruit quality and flavor, alongside the decline of defensive alkaloids. In parallel, fruit-associated bacterial communities undergo pronounced succession, with increasing diversity and a shift from Gram-negative, consumer oriented taxa towards Gram-positive and metabolite-associated genera in red ripe fruits. The strong correlations observed between enriched bacterial ASVs and key ripening metabolites suggest that fruit chemistry and microbiome composition are closely intertwined during maturation. Although correlative, these patterns indicate functional differentiation among microbial taxa occupying distinct ripening niches. Overall, our findings advance understanding of tomato fruit ripening as a coupled metabolic-microbial process and highlight the fruit microbiome as a potential target for improving fruit quality, flavor development, and postharvest management.

## Data Availability

All the raw sequencing data of bacterial communities associated with fruits of the cherry tomato from this project are available in the NCBI Sequence Read Archive (SRA) database under BioProject PRJNA1400201.
