# Supplementary material for: From green to red: metabolic reprogramming and bacterial community succession underpin cherry tomato fruit ripening and quality formation
Source: Front Plant Sci. 2026 Jun 8;17:1843442. doi: 10.3389/fpls.2026.1843442 (PMC13284048; doi:10.3389/fpls.2026.1843442)
Supplement: Supplementary file 1 [file Table1.docx]

**From Green to Red: Coordinated Metabolic Reprogramming and Microbiome Succession Underpin Cherry Tomato Fruit Ripening and Quality Formation**

Muzammil Hussain^1,2,3,*^, Nazir Ahmed^1,2,3^, Zhengzhou Yang^1,2,3^, Xiaona Xie^1,2,3^, Wenjing Xing^1,2,3^, Hongzhu Su^1,2,3^, Qingqing Peng^1,2,3^, Zhengjie Zhu^1,2,3,^*

^1^ College of Agriculture and Food Engineering, Baise University, Baise 533000, China

^2^ Guangxi Key Laboratory of Biology for Mango, Baise 533000, China

^3^ College of Subtropical Characteristic Agricultural Industry, Baise, China

^†^These authors have contributed equally to this work

Corresponding author’s email: muzammil0991@gmail.com (M. Hussain)

zhuzhjie@bsuc.edu.cn (Z. Zhu)

ORCID: 0000-0001-6681-7513 (M. Hussain)


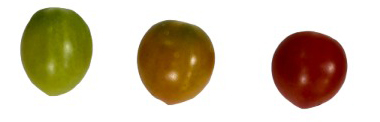


Figure S1. Representative stages of cherry tomato fruit ripening showing the progression from mature green (left), pink stage (middle), to fully red ripe stage (right).


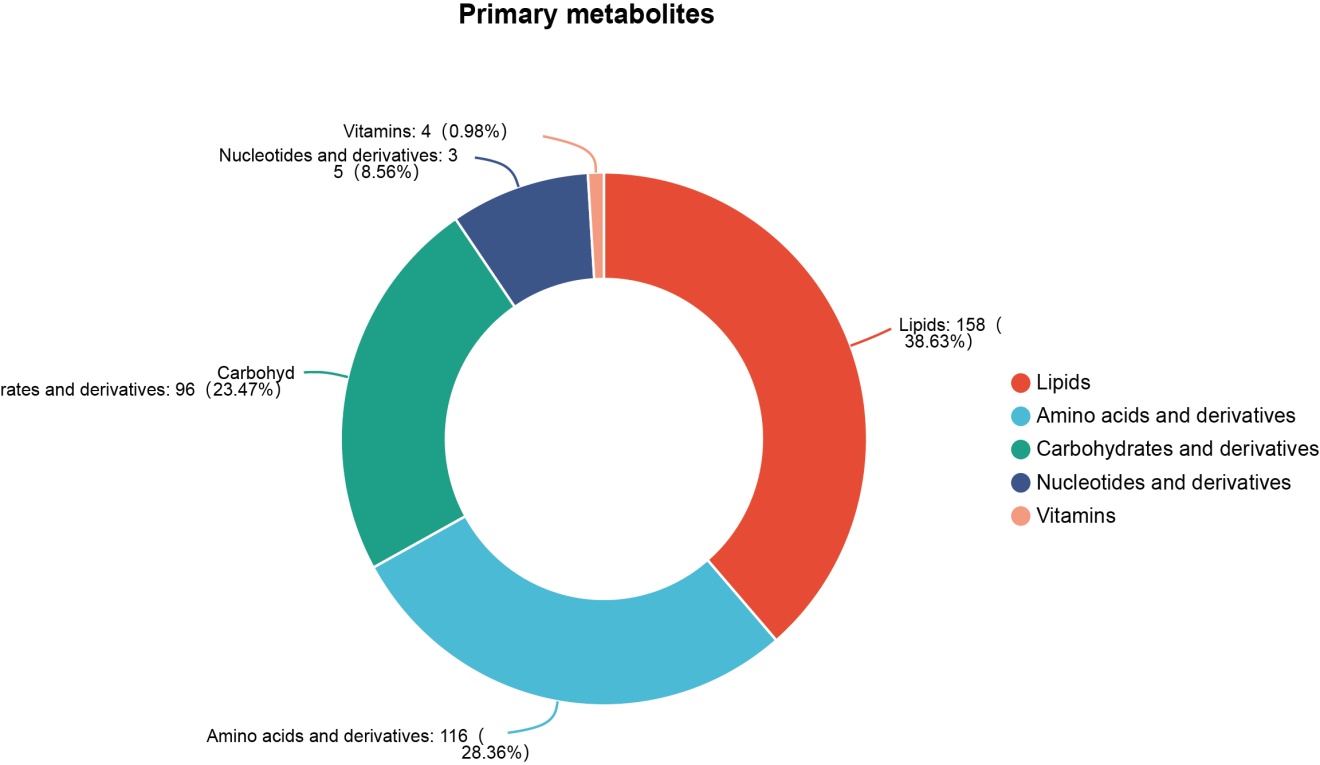


Figure S2: The composition of primary metabolites during cherry tomato fruit ripening.


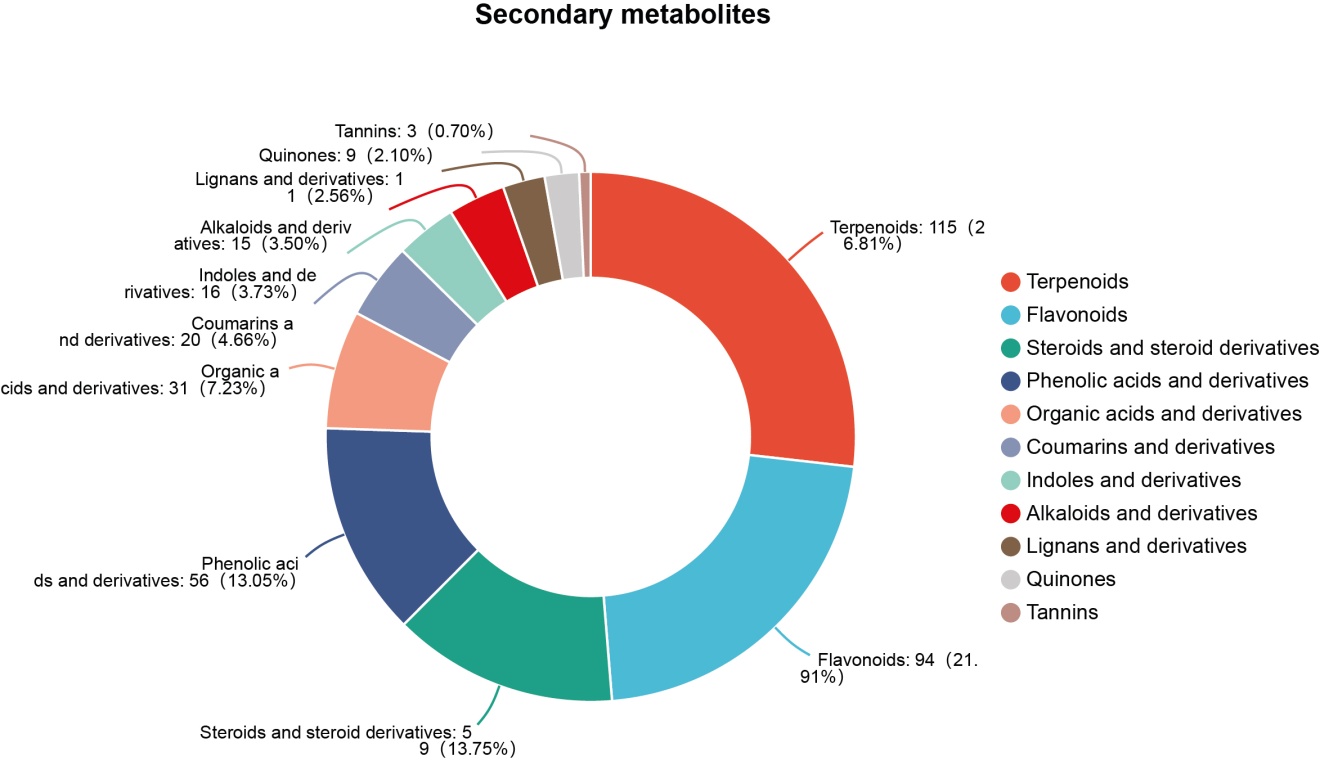


Figure S3: The composition of secondary metabolites during cherry tomato fruit ripening.


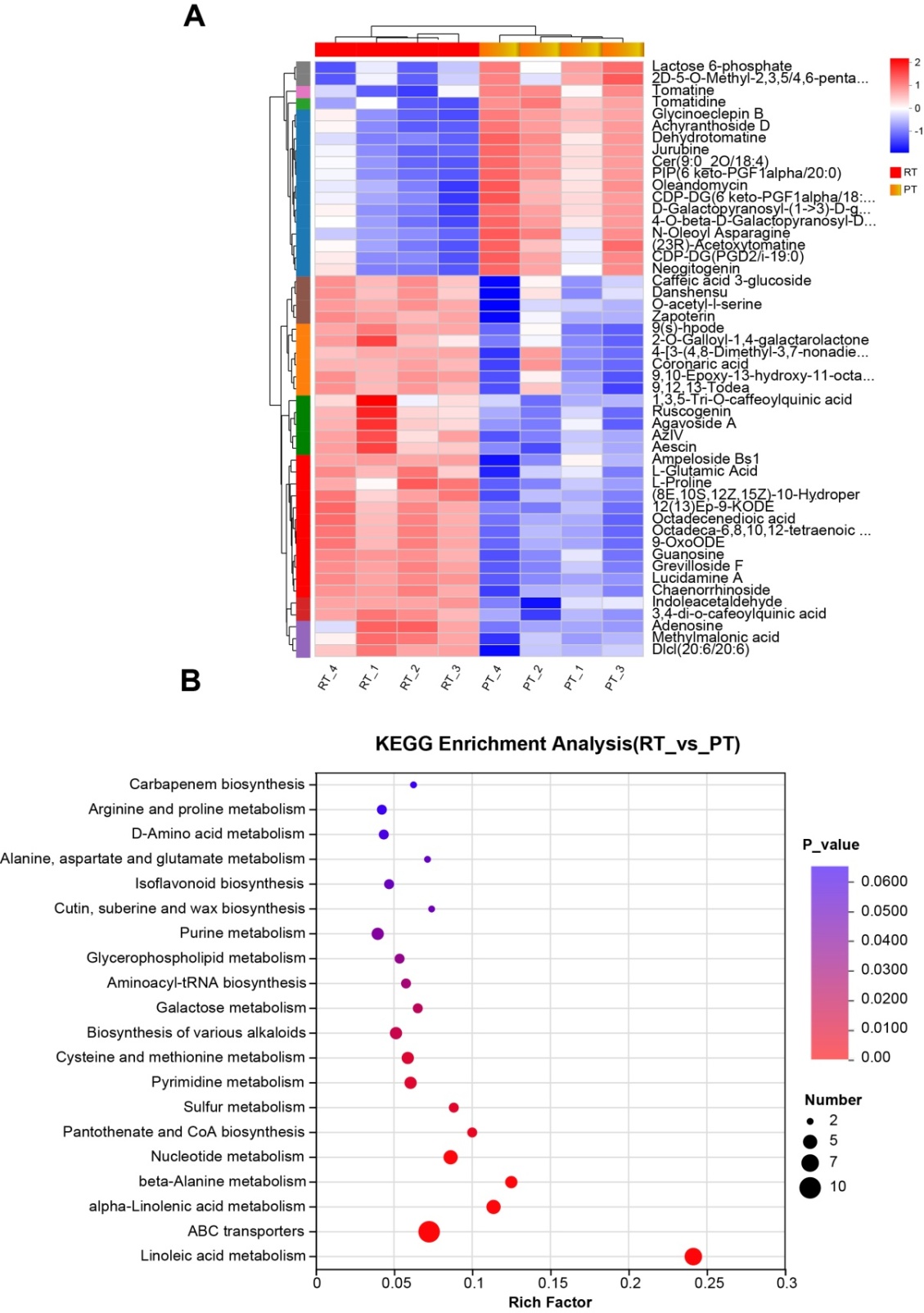


Figure S4: Metabolites profiling of cherry tomatoes at different ripening stages. (A) Heatmap of top 50 significantly differential metabolites in RT and PT samples, where red represents higher expression and blue represents lower expression. (B) KEGG enrichment analysis highlighting the significantly enriched metabolic pathways in RT compared to PT.


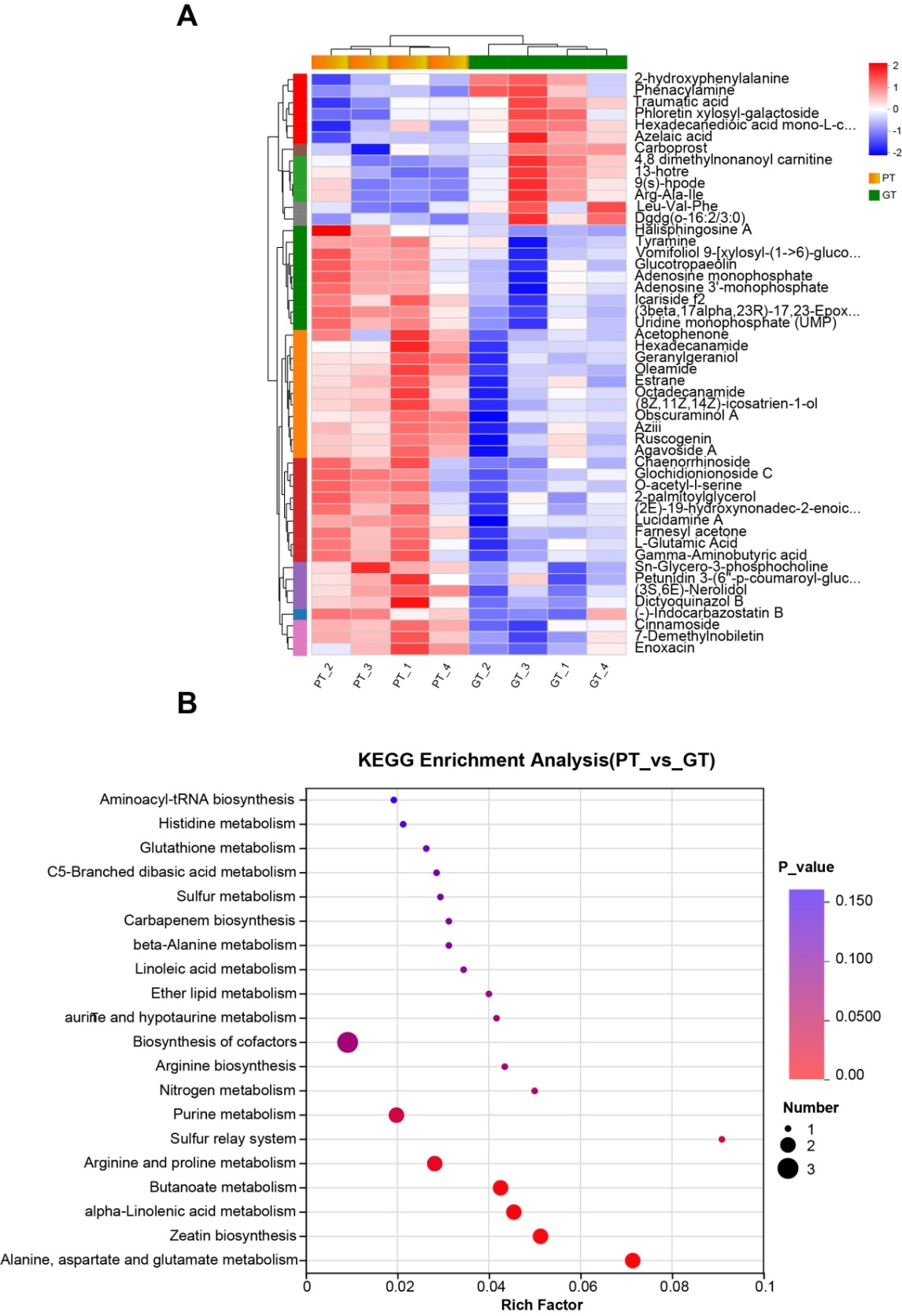


Figure S5: Metabolites profiling of cherry tomatoes at different ripening stages. (A) Heatmap of top 50 significantly differential metabolites in PT and GT samples, where red represents higher expression and blue represents lower expression. (B) KEGG enrichment analysis highlighting the significantly enriched metabolic pathways in PT compared to GT.


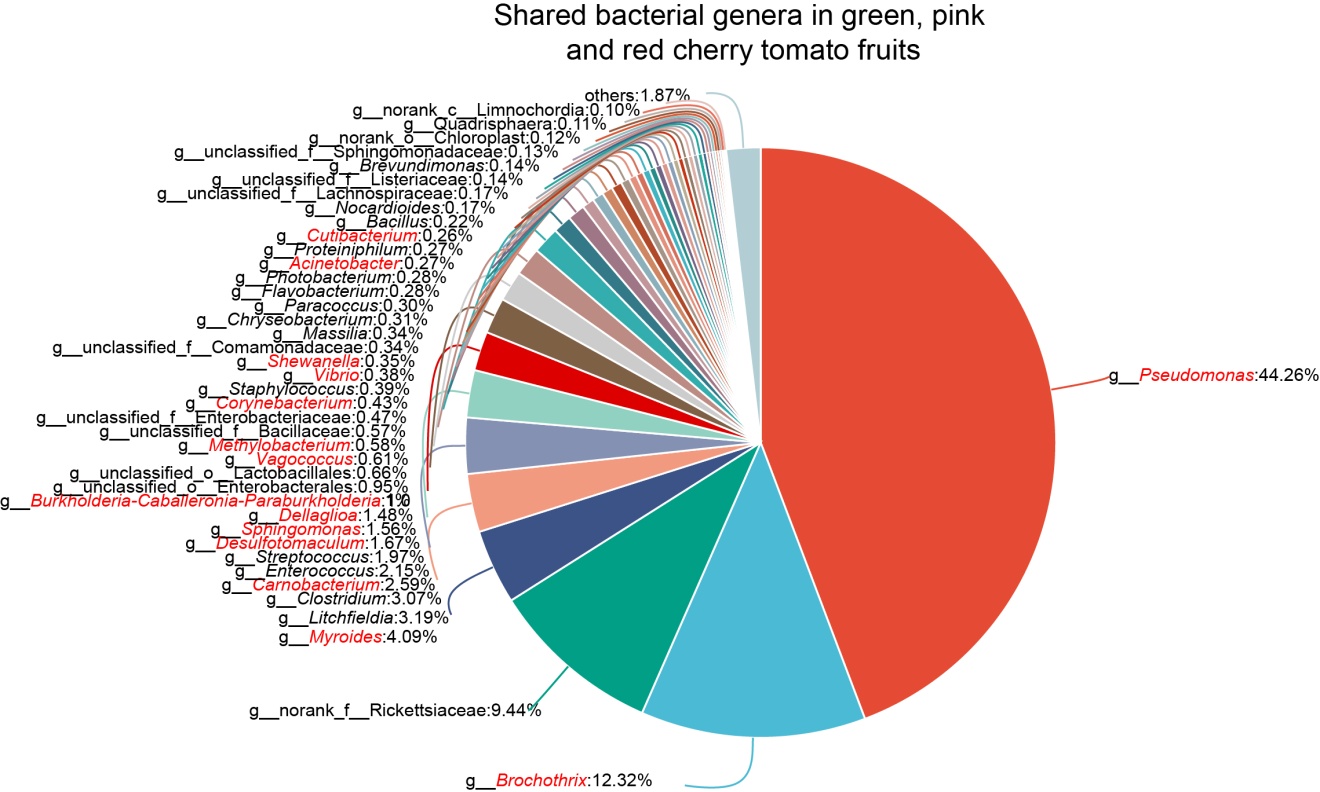


Figure S6: The shared bacterial taxa across the cherry tomato ripening stages at the genus level are depicted in the pie chart. Core bacterial genera are highlighted in red font in the pie chart.


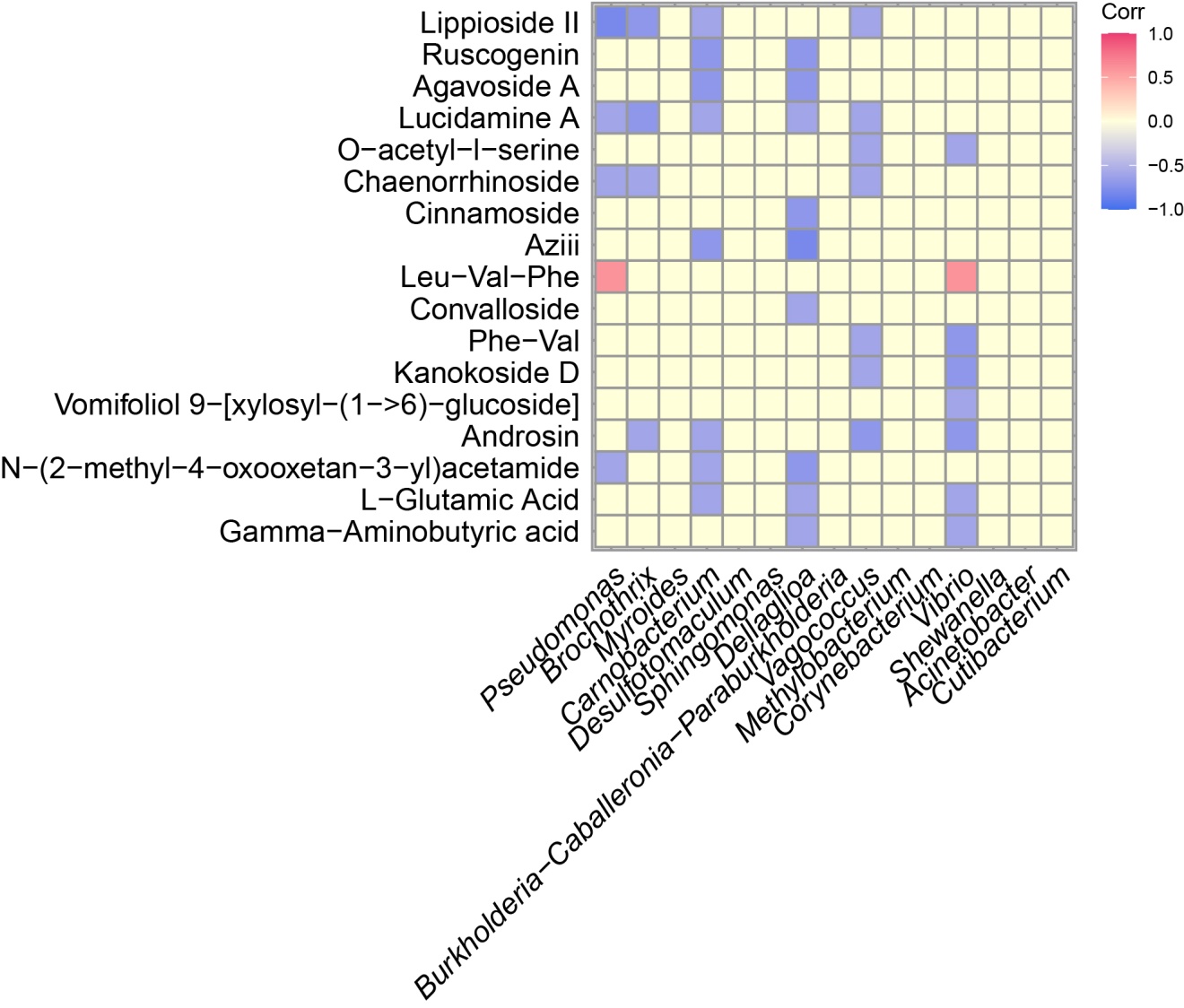


Figure S7: Correlation heatmap showing the relationships between the fruit-associated core bacterial genera and metabolites in cherry tomato fruits. Red color boxes indicate positive correlation, while blue boxes indicate negative correlations.

Table S1. Taxonomic information of bacterial ASVs significantly enriched between different ripening stages: GT vs PT, GT vs RT, and RT vs PT as shown in Fig. 6 (B-D). The ASVs are labeled by their respective genera (g) or family (f) or order (o) based on taxonomic annotation.

| **GT vs. PT** | | |
| --- | --- | --- |
| **ASVs** | **Taxonomic classification** | **Enriched in GT or PT** |
| ASV606 | g__*Pseudazoarcus* | PT |
| ASV22 | o__Peptostreptococcales | GT |
| ASV610 | g__*Levilactobacillus* | PT |
| ASV101 | g__*Pseudomonas* | PT |
| ASV605 | g__*Paracoccus* | PT |
| ASV619 | g__*Burkholderia-Caballeronia-Paraburkholderia* | PT |
| ASV615 | g__*Parazoarcus* | PT |
|  | | |
| **GT vs. RT** | | |
| **ASVs** | **Taxonomic classification** | **Enriched in GT or RT** |
| ASV316 | g__*Streptococcus* | RT |
| ASV313 | g__*Enterococcus* | RT |
| ASV320 | g__unclassified_f__Enterobacteriaceae | RT |
| ASV324 | g__*Lactiplantibacillus* | RT |
| ASV322 | g__*Clostridium* | RT |
| ASV332 | g__*Bacillus* | RT |
| ASV354 | g__*Fonticella* | RT |
| ASV329 | g__norank_f__Peptococcaceae | RT |
| ASV351 | g__*Enterococcus* | RT |
| ASV343 | g__*Escherichia-Shigella* | RT |
| ASV605 | g__*Paracoccus* | RT |
| ASV376 | g__*Pediococcus* | RT |
| ASV325 | g__*Clostridium* | RT |
| ASV328 | g__norank_f__Peptococcaceae | RT |
| ASV333 | g__*Clostridium* | RT |
| ASV327 | g__*Clostridium* | RT |
| ASV314 | g__*Clostridium* | RT |
| ASV318 | g__*Litchfieldia* | RT |
| ASV8 | g__*Pseudomonas* | GT |
| ASV414 | g__*Flavobacterium* | RT |
| ASV330 | g__unclassified_f__Bacillaceae | RT |
| ASV315 | g__norank_f__Peptococcaceae | RT |
| ASV15 | g__*Pseudomonas* | GT |
| ASV336 | g__norank_f__Peptococcaceae | RT |
| ASV29 | g__norank_f__Mitochondria | GT |
| ASV101 | g__*Pseudomonas* | RT |
| ASV447 | g__unclassified_f__Alcaligenaceae | RT |
| ASV23 | g__unclassified_f__Listeriaceae | GT |
| ASV331 | g__*Clostridium* | RT |
| ASV32 | g__*Litchfieldia* | RT |
|  | | |
| **RT vs. PT** | | |
| **ASVs** | **Taxonomic classification** | **Enriched in RT or PT** |
| ASV313 | g__*Enterococcus* | RT |
| ASV22 | o__Peptostreptococcales | RT |
| ASV320 | g__unclassified_f__Enterobacteriaceae | RT |
| ASV331 | g__*Clostridium* | RT |
| ASV342 | g__*Romboutsia* | RT |
| ASV354 | g__*Fonticella* | RT |
| ASV332 | g__*Bacillus* | RT |
| ASV351 | g__*Enterococcus* | RT |
| ASV325 | g__*Clostridium* | RT |
| ASV343 | g__*Escherichia-Shigella* | RT |
| ASV322 | g__*Clostridium* | RT |
| ASV329 | g__norank_f__Peptococcaceae | RT |
| ASV327 | g__*Clostridium* | RT |
| ASV323 | g__*Clostridium* | RT |
| ASV321 | g__*Clostridium* | RT |
| ASV326 | g__*Desulfonispora* | RT |
| ASV39 | g__unclassified_f__Bacillaceae | RT |
| ASV376 | g__*Pediococcus* | RT |
| ASV56 | g__unclassified_f__Peptostreptococcaceae | RT |
| ASV333 | g__*Clostridium* | RT |
| ASV328 | g__norank_f__Peptococcaceae | RT |
| ASV314 | g__*Clostridium* | RT |
| ASV317 | g__*Litchfieldia* | RT |
| ASV318 | g__*Litchfieldia* | RT |
| ASV315 | g__norank_f__Peptococcaceae | RT |
| ASV330 | g__unclassified_f__Bacillaceae | RT |
| ASV336 | g__norank_f__Peptococcaceae | RT |
| ASV8 | g__*Pseudomonas* | PT |
| ASV29 | g__norank_f__Mitochondria | PT |
| ASV615 | g__*Parazoarcus* | PT |
| ASV507 | g__norank_f__Mitochondria | RT |
| ASV338 | g__*Clostridium* | RT |
| ASV606 | g__*Pseudazoarcus* | PT |
| ASV32 | g__*Litchfieldia* | RT |
| ASV324 | g__*Lactiplantibacillus* | RT |
